# Characteristics of antenna fabricated using additive manufacturing technology and the potential applications

**DOI:** 10.1016/j.heliyon.2024.e27785

**Published:** 2024-03-15

**Authors:** Muthanna Aziz, Amged El Hassan, Mousa Hussein, Essam Zaneldin, Ali H. Al-Marzouqi, Waleed Ahmed

**Affiliations:** a Mechanical and Aerospace Engineering Department, College of Engineering, UAE University, United Arab Emirates; b Electrical Engineering Department, College of Engineering, UAE University, United Arab Emirates; c Civil and Environmental Engineering Department, College of Engineering, UAE University, United Arab Emirates; d Chemical and Petroleum Engineering Department, College of Engineering, UAE University, United Arab Emirates; e Engineering Requirements Unit, College of Engineering, UAE University, United Arab Emirates

**Keywords:** 3D printing, Antenna, Fabrication, Characteristics

## Abstract

Antennas play a critical role in modern technology. They are used in various devices and applications, including wireless communication, broadcasting, navigation, military, and space. Overall, the importance of antennas in technology lies in their ability to transmit and receive signals, allowing communication and information transfer across various applications and devices. Three-dimensional printing technology creates antennas using multiple materials, including plastics, metals, and ceramics. Some standard 3D printing techniques used to create antennas include Fused Deposition Modeling (FDM), Stereolithography (SLA), and Selective Laser Sintering (SLS). These antennas can be made in various shapes and sizes. 3D printing can help create complex and customized antenna designs that are difficult or impossible to produce using traditional manufacturing methods. 3D-printing technology has many advantages for building antennas, including customization, ease of fabrication, and cost-effectiveness. This review comprehensively evaluates the usage of 3D-printing technology in antenna fabrication.

## 1 Introduction

Three-dimensional (3D) printing is a highly adaptable technology that is increasingly utilized with other technologies to create innovative and cost-effective products. The additive manufacturing (AM) process involves layering tiny layers of material together to generate a 3D computer-aided design (CAD) model. On the contrary, traditional manufacturing is dominated by subtractive production. Subtractive manufacturing refers to removing or subtracting materials to form the final product. 3D-printing technology has taken hold of various industries, including the medical, electrical, and industrial fields. Electrical components and products are often produced using 3D-printing technology, bringing significant advantages. These devices were either previously unavailable, or making them using 3D printing was highly expensive compared to building them using other techniques. The 3D-printing technology is primarily based on two essential factors: 1) the technical features required for 3D-printing materials and 2) the development of materials used by the 3D-printing process. These fundamental aspects are thought to be the industry basis. Hence, materials used by the 3D-printing technology have become an essential factor in the industry's growth due to their wide range of applications, all of which directly affect the evolution of the applied technology. The different types of nanomaterials utilized in the 3D-printing technology in the electrical and biological domains are covered in this review.

Furthermore, the benefits of 3D printing technology and their influence on the quality of objects made using 3D printing are investigated. Various commercial uses of 3D printing materials are discussed to demonstrate the technology's industrial influence. Most engineers and researchers working in the field of 3D printing focus on materials such as ceramics and biomass, as well as biocompatible and antibacterial materials. Antennas and electromagnetic structures are printed using various 3D-printing processes like Fused deposition modeling (FDM), fused filament fabrication, Stereolithography (SLA), micro-SLA, polyjet printing, and photonic polymerization. Each approach has benefits and drawbacks appropriate for various types of antennas. For example, FDM is the most affordable 3D-printing technology. It provides an extensive range of filaments and can print with multiple materials in a single print. Laser or UV light preserves resin components in an appropriate 3D form in the SLA and polymerization processes. As a result, it can achieve a far higher resolution (down to the size of tens of nanometers) than FDM. It is used to create antennas in the millimeter-wave and THz frequency bands because it is more suited for creating tiny structures. The materials used in these printers cannot be modified as much as the FDM filaments because they are essentially light-curable resin. 3D printing is the most commonly used technology to produce dielectric-based antennas. Creating dielectric materials is considerably easier than creating conductive filaments, which explains why FDM can be used to develop dielectric materials.

## 2 Microstrip patch antenna

A wideband antenna with a two-dimensional (2D) physical geometry is formed using a planar metal sheet or a “patch,” which may be rectangular, circular, triangular, or any other shape, and placed on one side of an insulating dielectric substrate on the other side. This conductive layer serves as a ground plane is attached. This antenna has a low profile, is inexpensive, can be easily fabricated, and has high conformability. It is typically utilized in microwave applications and offers more design flexibility regarding bandwidth compared to other antennas, such as microstrip dipole and microstrip slot antennas.

AM is commonly used to produce microstrip patch antennas owing to their simple geometrical structure and ease of fabrication. Several 3D printing technologies were utilized in the fabrication of Microstrip antennas. In Refs. [1,2], FDM was utilized to print an antenna's conducting and substrate layers. Only the nonconductive substrate was fabricated through FDM in Refs. [[3], [4], [5], [6]]. Screen printing was also reported in Refs. [[7], [8], [9]], in addition to using inkjet 3D printing in Refs. [10,11]. Two 3D printing processes, i.e., stereolithography (SLA) and inkjet printing, were employed to fabricate the patch antennas [12,13].

**Fig. 1:**
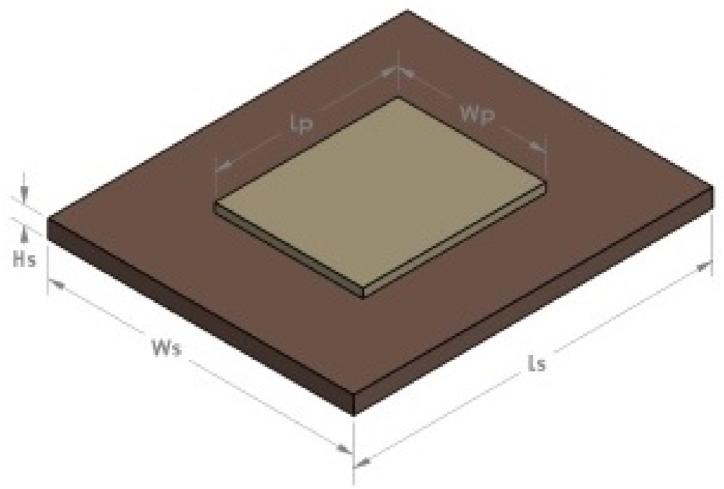
3D CAD model of the patch antenna [1].

**Fig. 2:**
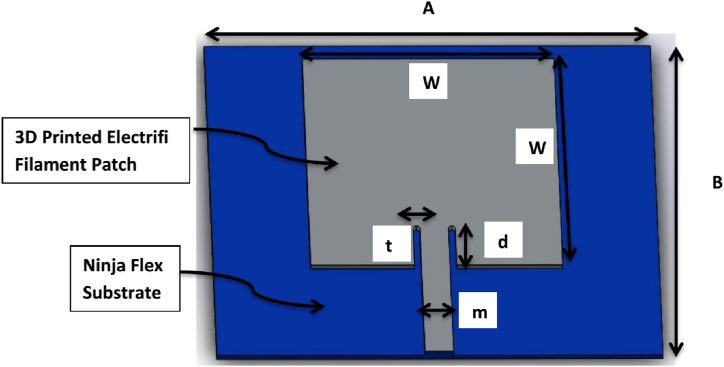
3D CAD model of the conformal patch antenna [2].

Hasni et al. [1] investigated a single-step 3D-printing approach for fabricating the patch antenna. The dielectric and conductive parts of the antenna were 3D-printed in a single step employing the dielectric and conducting filaments and avoiding the need for a second phase involving conductive ink extrusion and curing. They examined how the tool path and layer resolution affected the complex permittivity and conductivity of the substrate and the radiative materials. This approach may be a cost-effective replacement for traditional microwave antenna construction procedures in non-conventional circumstances. Mitra et al. [2] presented a 3D-printed conformal microstrip patch antenna. The antenna's flexible conducting layer and flexible nonconductive substrate were 3D-printed using the FDM technology. Fig. 1 shows that a 5.8 GHz probe-fed patch antenna was then designed in HFSS. Patch antenna, also known as a rectangular microstrip antenna, was selected due to its ease of manufacture and balun-less topology, and Fig. 2 depicts the representations of the 3D CAD model of Prototype of the 3D-printed microstrip patch antenna with conductive Electrifi filament on NinjaFlex substrate modeled in the full-wave tool Ansys HFSS. All dimensions are in mm. (A = 65.55, B = 55.5, W = 37, d = 7, t = 1, and m = 3).

Prakash et al. [3] proposed a 3D-printed substrate for the microstrip patch antennas fabricated through FDM. Conductive copper paint was applied on the 3D-printed PLA substrate to form the radiating patch and ground plane. The theoretical and simulation results were comparable. Based on the results, the FDM-printed components can be used as a substrate for producing microstrip patch antennas. Belen and Mahouti [4] studied the creation of a broadband microstrip quasi-Yagi antenna composed of a ground reflector, a patch with a rectangular form, and a dipole supplied via a coplanar strip line. FDM was used to produce the substrate. Copper tape was then applied to the top and ground layers. They aimed to operate an antenna for indoor applications within the 670–3000 MHz operating frequency range. Sanchez‐Olivares et al. [5] demonstrated a circular conformal array antenna of a double-stacked microstrip patched in two separate radiations. The first was an omnidirectional beam. The second was an electronically switched directed beam. The conformal holding structure and the microstrip devices were fabricated using the PCB and FDM 3D-printing technology, which considerably lowered the cost and weight of the prototypes.

MacDonald et al. [6], described a 3D-printed patch antenna that was embedded in a complex dielectric structure related to aerospace isogrid panels. They used the AM technique, called hybrid multiprocess, that combines the extrusion of polymer materials with supplementary production skills, including foil insertion, patterning, wire integration, and component placement. The porosity and unstable mechanical connection between the output connector and the substrate were two fabrication issues mentioned by the authors. Porosity affected the substrate's dielectric properties, leading to final antenna resonance errors. An unreliable mechanical connection caused Variations in input impedance, which reduced the signal quality. Several solutions have been proposed, all involving junction strengthening using a bonding agent during embedding. The observed reflection and gain coefficients were acceptable compared to those of a standard dipole antenna. Ram et al. [7] proposed a prototype that combined a printed patch-antenna structure with a solar panel while retaining the optical efficiency to act as both an antenna and a solar panel. Through screen printing, a graphene-based CPW-fed E-shaped microstrip patch antenna was fabricated on a solar panel. Its properties were then analyzed and discussed.

In conclusion, the proposed design approach is particularly suited for satellites because it reduces the space complexity of the satellite system by one-third of its initial dimensions. Ram et al. [8] presented a multilayergraphene–based, screen-printed flexible microwave antenna that uses paper and polystyrene because they are more readily available and less expensive than the flexible substrate. The antenna was tested to see how multilayer printing would affect its performance, and it was successful. Meanwhile, Abhilash et al. [9] discussed the creation of a patch antenna using a novel LTCCglass–ceramic composite material created using a specific quench-free glass. Sr_2_ZnTeO_6_ (SZT) and 5% ZBPT (10 mol% ZnO–2 mol% B_2_O_3_–8 mol% P_2_O_5_–80 mol% TeO_2_) glass made up this unusual composition, with the latter reaction mixture being added straight to the ceramic and skipping the higher temperature melt quenching stage. The radiation characteristics of a ceramic patch antenna were also exhibited. Matyas et al. [10] described a method that can be utilized to construct a flexible microstrip antenna via the inkjet 3D-printing technology. The antenna was fabricated using silver nanoparticles on a flexible PET foil.

The nanoparticles were made using the solvothermal precipitation method. Goh et al. [11] investigated low-cost, rapid-production solutions for manufacturing patch-antenna emitters. They also used the AM technology to create two patch antennas using silver nanoparticle ink on an FR4 substrate. The suggested approach offers a commercially feasible option for producing emitters and antennas using inkjet printing. Heirons et al. [12] merged two separate processes for fabricating circularly polarized patch antennas using 3D-printing technology, where SLA and inkjet printing were used. They also conducted experimental and simulation studies and investigated whether these simulation results were in good agreement with the practical effects. Jun et al. [13], performed 3D-printed circularly polarized patch-antenna fabrication using a combination of inkjet printing and SLA. They used curing photosensitive resin to print the substrate and silver ink to create the conductive patch element.

Furthermore, they utilized simulation and experimental findings to analyze the performance with the excellent results achieved. Wang et al. [14] described the use of a 3D-printed substrate with an inherent asymmetry to create a microstrip patch antenna with circular polarization. They also investigated the possibility that the substrate asymmetry, where a pair of triangular air inserts was symmetrically etched in one of the diagonals of the square substrate, was the origin of the disturbance for creating CP waves. Lastly, Yang et al. [15] investigated the effect of the laser-sintering factors on the sintering quality of themetal–organic decomposition ink with a silver component. A mathematical model was established to disclose the temperature evolvement in MOD inks and substrates during laser sintering. The simulation and experimental findings revealed that the laser-sintering factors substantially affected the sintering quality. A microstrip antenna was finally produced using the optimized sintering approach. The antenna's performance aligned with the simulation, confirming the effectiveness of the optimizing method.

Table 1 presents the specifications for the microstrip patch antenna fabricated through 3D printing.

**Table 1:** Specifications of the 3D-printed microstrip patch antenna.

Ref.	Freq	Antenna type	Fabrication technique and materials	Application	Advantage	Limitation
[1]	SHF	Patch antenna	Graphene-based FDM-conductive PLA filament (Black Magic) was used as the material for the ground plane and radiating components. Polylactide acid (PLA) for the substrate is dielectric	Microwave applications	The dielectric and conductive portions of antennas are 3D-printed using dielectric and conducting filaments in a single-step fabrication process, thereby eliminating the need for a second step involving conductive ink extrusion and curing. A possible cost-effective replacement for traditional microwave antenna fabrication processes in unconventional environments	Significant losses in the conductive medium were discovered, resulting in shallow gain values.
[2]	UHF	Conformal patch antenna	Fused filament fabrication. Electrify on a NinjaFlex substrate	Terrestrial Wi-Fi systems, DoD applications, and flexible and wearable applications	A conformal microstrip patch antenna can be made entirely via additive manufacturing (AM).	Higher cost in comparison to commercial adhesive copper foil made from a flexible NinjaFlex substrate with the top and bottom conductive layers of Electrify filament.
[3]	SHF	Microstrip conformal patch antenna	FDM PLA	WLAN and C-band, spacecraft, satellite, aircraft, and wireless communication systems	High performance, compact size, and easy to install. Fabrication time and cost are reduced as multiple machining processes are avoided. Using the PLA substrate results in an increased number of resonant bands and good impedance matching.	Fabrication error and connection loss are to blame for the discrepancy between the measured and simulated values.
[6]	UHF	Patch antenna	FDM (multiprocess AM) Polycarbonate (PC) filament, copper foil for the radiating element and ground plane	Portable wireless devices and space vehicle applications	Competing performance compared to the dipole antenna. Antennas may be included in multipurpose panels to increase volumetric effectiveness and reduce weight.	Due to porosity, the goal frequency was not achieved and was 11.5% less (2.4 GHz target with 2.14-GHz actual resonance). Input impedance changes caused by the unstable mechanical connection between the output connector and the substrate reduced the signal quality.
[7]	UHF	Microstrip patch antenna	Screen printing Graphene nanomaterial ink	(Satellites) microwave applications	Combining a solar panel and a patch-antenna structure makes it possible to combine the functions of an antenna and a solar module while reducing the space complexity of the satellite system by one-third of its original dimensions.	Due to the semitransparent quality of the graphene conductive ink, there is a 9% decrease in the efficiency of the solar panel.
[9]	UHF	Microstrip patch antenna	Screen printing on a newly developed LTCC tape Silver ink	Microelectronic and microwave communication, as well as hard ceramic substrate applications	Lightweight, consistent gain, consistent radiation pattern, low cost, and compatibility with compact portable wireless device circuits	The deviation in radiating frequency may occur due to the connector's influence. Furthermore, minute changes in the machining antenna dimensions, surface uniformity of the printed radiating pattern, and even geographical inhomogeneities in the dielectric properties of the ceramic substrate should be considered.
[10]	UHF	Flexible microstrip antenna	Inkjet printing Silver nanoparticles are printed on a flexible polyethylene terephthalate foil.	Wearable electronic devices	Flexible and low-weight	The printed layer's homogeneity significantly impacts the quality of the resulting microstrip antenna. On average, achieving excellent homogeneity of thin layers printed using inkjet technology is challenging.
[11]	SHF	Microstrip patch antenna	Inkjet printing Silver nanoparticles ink on FR4 substrate	Wireless communication applications	Cost reduction, fast and simple solution to design and print microstrip patch antennas, lightweight and compact	Silver is an undesirable option for mass manufacturing due to its high cost. Although copper and aluminum are less expensive options, they oxidize readily in the environment and produce nonconductive oxides. The most attractive and promising alternative to metal-based inks is graphene.

## 3 Dielectric resonator antennas

Compared to the microstrip patch antenna, the dielectric resonator antenna (DRA) offers superior efficiency, particularly at the millimeter-wave frequency, due to the lower propagation losses and larger fractional bandwidth. This advantage makes it an attractive replacement for the microstrip patch antenna at higher frequencies. FDM 3D printing is mainly used to fabricate the DRA antennas.

**Fig. 3:**
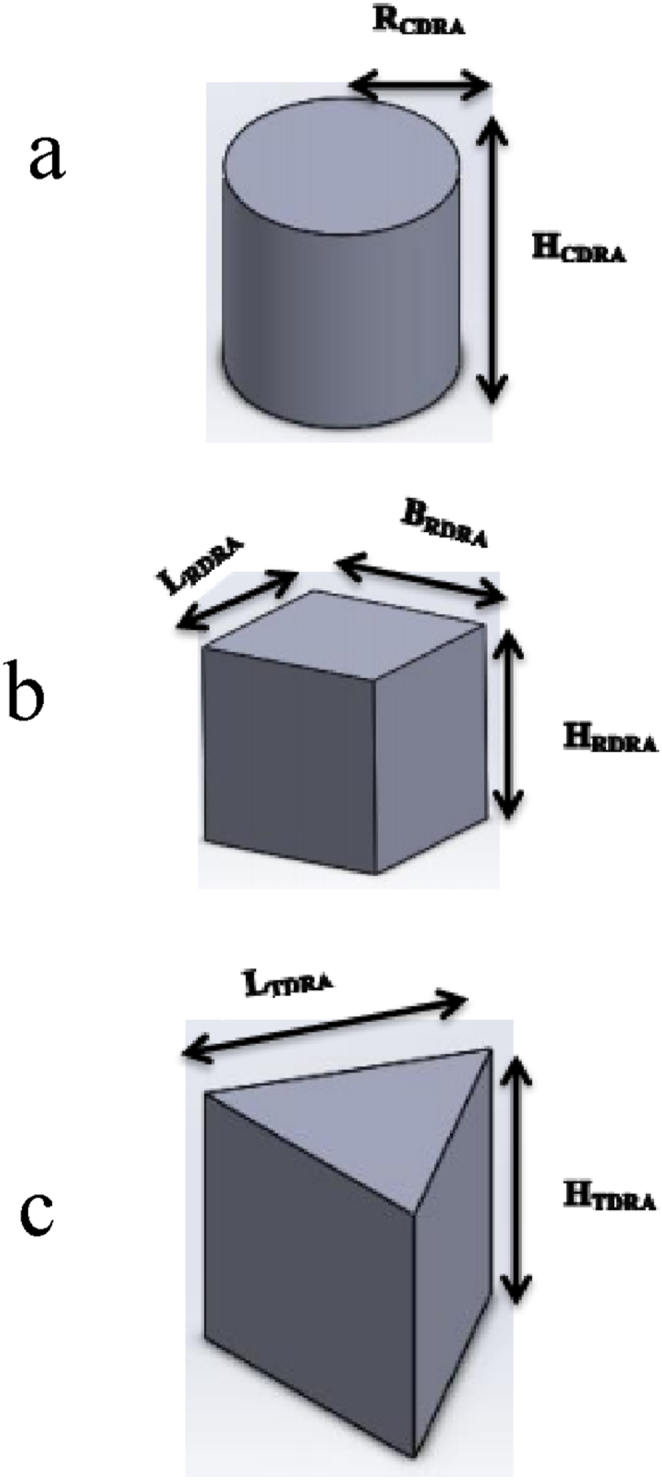
3D CAD model of the radiator structure [16].

Kumar et al. [16] demonstrated the feasibility of fabricating a 3D radiator structure using biodegradable polylactic acid (PLA). They produced and studied three conventional DRAs, namely, the cylindrical, rectangular, and triangular dielectric resonator antennas, and showed that their antennas are compact, lightweight, and cost-effective. The suggested antennas may be used for weather monitoring, air traffic control, scanning and discrimination, mobile services, and other wireless C-, X-, and Ku-band applications. The design and optimization of the coaxial feed structure are carried out in such a way that it operates in a non-resonating mode as shown in Fig. 3 Proposed Dielectric Resonator Antenna (DRAs) geometry: (a) Cylindrical DR Antenna (CDRA), (b) Rectangular DR Antenna (RDRA), and (c) Triangular DR Antenna (TDRA).

Wu et al. [17] employed characteristic mode analysis (CMA) to examine a dielectric antenna produced via 3D printing. They presented a CMA formulation for a 3D-printed antenna with the feeding and modal resonances, Q factor, and power factor, which might be helpful for future antenna analysis and improvement. Meanwhile, Kumar et al. [18] studied and presented a portable multiwavelength antenna for UWB wireless data links, an electromagnetic sensor, and multibeam applications. The antenna structure combined an air-suspended rectangular DRA and a printed horn. The DRA was 3D printed using PLA and held in place in the air by four dielectric pillars. The proposed antenna exhibited multibeam characteristics and a good performance at different frequencies. Liu et al. [19] used 3D printing to create a single-feed, dual-band, circularly polarized, and reconfigurable liquid DRA. They developed a dual band by exciting the quasi-TE 111 and TE 113 modes of the rectangular LDRA. According to the measured data, the proposed antenna may achieve CP reconfigurability in dual bands with broad bandwidths, including GPS and wireless local area network (WLAN) bands. Belen et al. [20] proposed a DRA combined with a frequency selective surface for ISM band applications that were simple to prototype, lightweight, inexpensive, and high in performance. With the measured performance, the proposed antenna was smaller than the alternative antenna designs in the literature.

Table 2 outlines the specifications pertaining to the dielectric resonator antennas.

**Table 2:** Specifications of the dielectric resonator antennas.

Ref.	Freq	Antenna type	Fabrication technique and materials	Application	Advantage	Limitation
[16]	SHF C band, X band, and Ku band	CDRA, RDRA, and TDRA	FDM PLA	Numerous C-, X-, and Ku-band wireless applications, including weather monitoring, radio navigation, air traffic control, scanning and discrimination, and mobile service	Compared to traditional ceramic DRA, 3D-printed DRA is smaller, lighter, easier to fabricate, has higher thermal conductivity, and is less expensive.	Thermal stability limitations. Variations in resonance frequency and bandwidth.
[17]	UHF	Conformal wire feeds the hollow sphere	FDM PLA	Using 3D printing as a new alternative for creating DRAs with variable forms, diverse permittivity and physical characteristics, great flexibility, and low-cost	High flexibility and low cost	Unfortunately, the Q factors of the dominant modes rise in the same order as wire length. As a result, matching the antenna and obtaining reasonable bandwidth becomes more challenging.
[18]	SHF UWB (7.5–31.3 GHz)	Portable multiwavelength DRA antenna	FDM PLA	Wireless datalink (omnidirectional and multi-beam) and biomedical applications, as well as electromagnetic sensors	Increased gain and reduced electromagnetic interference	The radiation effectiveness of the suggested antenna is limited up to 20 GHz, beyond which it begins to decline owing to the increasing material loss.

## 4 Horn antenna

**Fig. 4:**
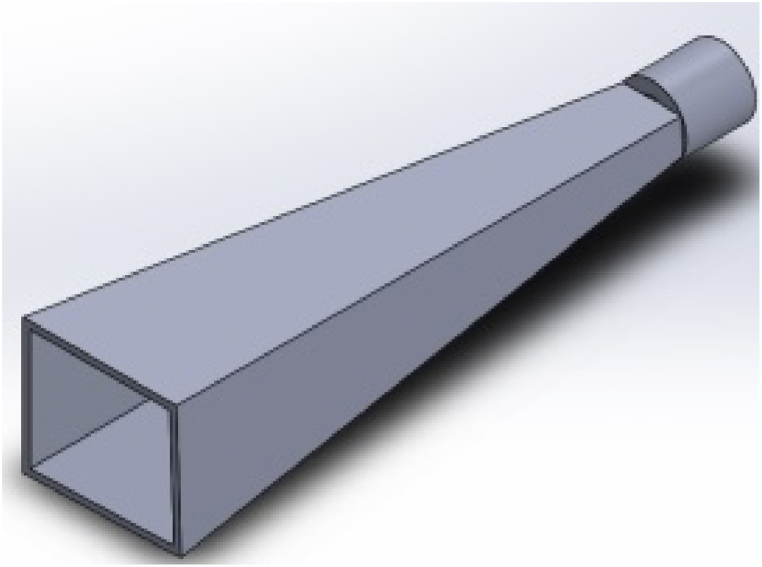
3D CAD model of the rectangular horn antenna [21].

AM allows rapid prototyping of new, complex 3D designs that are lighter and difficult to achieve with traditional fabrication techniques. Despite the benefits, surface roughness is one of the challenges reported when fabricating horn antennas using metallic 3D printing (e.g., selective laser melting (SLM)). Surface roughness has a significant influence on the antenna performance. To address the well-known issue of excessive surface roughness in 3D-printed components, Lamagna et al. [21] examined how two basic types of surface postprocessing affect the functionality of a rectangular 3D-printed antenna made using the SLM technique. Co-polar and cross-polar angular responses were compared throughout the W band in another experiment. They concluded that the W band can perform well with either postprocessing method. Fig. 4 depicts a rectangular horn antenna designed to reproduce the features of the commercially available Aerowave 08-7025 horn by feeding a standard WR8 rectangular waveguide.

**Fig. 5:**
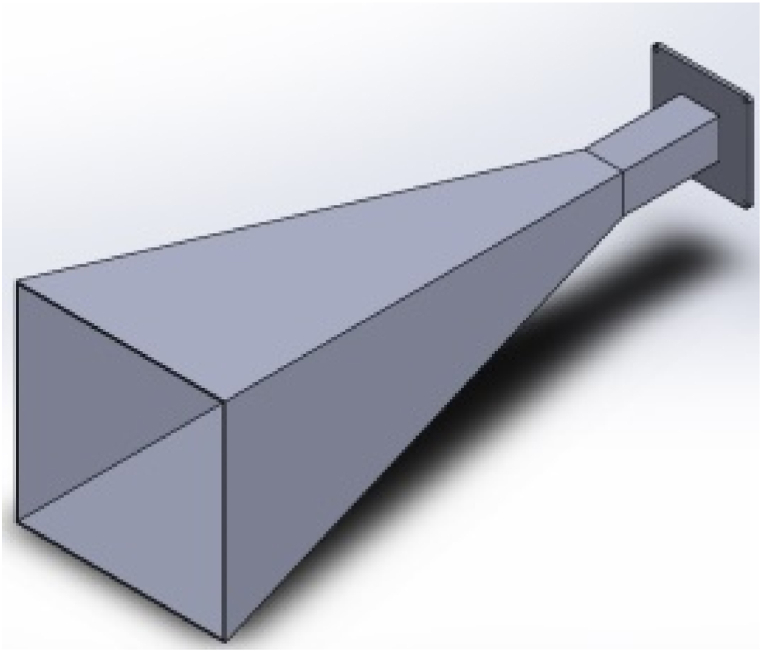
3D CAD model of the circularly polarized horn antenna [22].

Kotze and Gilmore [22] demonstrated an SLM 3D-printed circularly polarized horn antenna suitable for satellite communications in the X-band domain. Among the benefits are decreased weight, fewer materials, and the capability to build the antenna as a single solid component, eliminating the need for assembly and providing perfect electrical continuity. However, the reported limitations include surface roughness, metal oxidation, and the high cost of the fabrication method. Zhang et al. [23] fabricated K- and Ka-band polarization reconfiguration horn antennas using 3D metal–printing technology. They conducted simulation and theoretical experiments and validated the results. Fig. 5 presents the CAD model of the circularly polarized horn antenna.

**Fig. 6:**
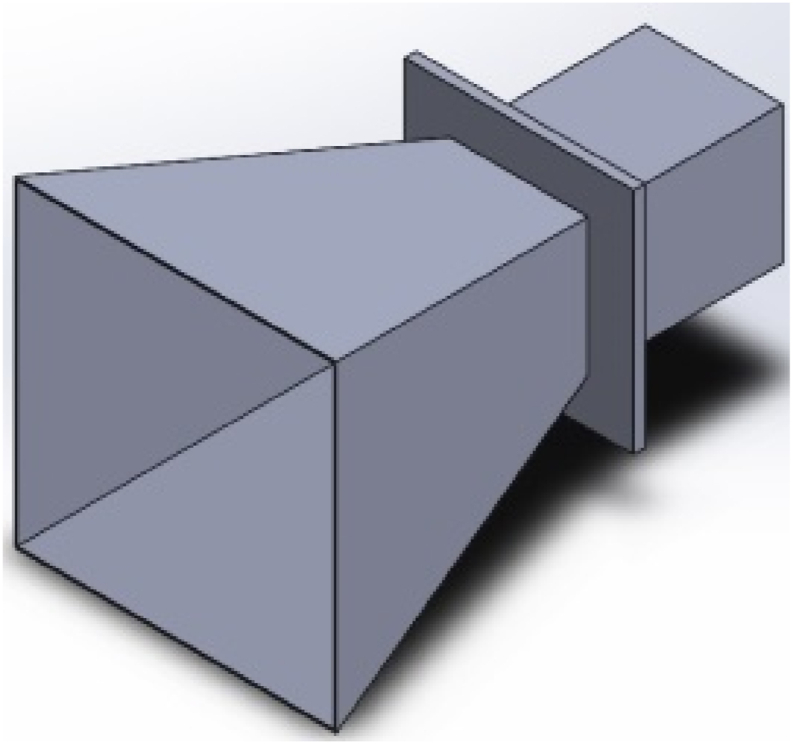
3D CAD model of the horn antenna [24].

An alternative approach for fabricating horn antennas combines two processes: 3D printing the antenna with a dielectric material and metalizing it. Kyovtorov et al. [24] created the antenna via 3D printing in a polymer and then metalized it via chemical and electrochemical deposition. They also compared a conventional metal pyramidal horn prototype to its 3D-printed replica. The results revealed that the proposed antenna performed very similarly to the reference metal prototype in the SHF band aside from being lightweight, robust, and compact, which could be critical for applications requiring complex geometries and/or low weight, such as UAVs. Bongard et al. [25] investigated the feasibility of using metal-plated 3D-printed plastic parts to produce waveguide-based antenna arrays. For this, they fabricated and tested a 20-GHz waveguide-fed horn array with dual circular polarization utilizing the combination of 3D printing and conventional machining. As a result, they observed a good performance. Fig. 6 depicts the horn antenna made of the original metal copper alloy and the plastic CAD ABS reproduction.

Hosseini and Dahmardeh [26] examined a compact-mode converter antenna at 12-GHz frequency, which was fabricated using the FDM 3D-printing technology and coated with a thin conductive layer. The increased cross-section of the antenna significantly decreased the possibility of an electrical breakdown. The simulation results agreed well with the experimentally measured results. Castro et al. [27] fabricated a triple-mode circular waveguide–corrugated horn antenna through precision machining using an aluminum block and a 3D-printing-based method from PC-acrylonitrile butadiene styrene (ABS). They created a single structure with three distinct modes, namely, TE11, TM01, and TE21. They also compared the aluminum and 3D-printed corrugated horn antennas and showed similar normalized radiation patterns. Tak et al. [28] used 3D printing and conductive spray painting to create a lightweight, quickly constructed X-band waveguide horn antenna. The proposed antenna is low-cost, light, and can be rapidly built using a simple manufacturing procedure. The performance of the horn antenna was compared with that of an aluminum horn antenna of the same size. Wang et al. [29] presented a K-band antenna array by deposing the quasi-pyramidal horn print circuit board and forming a quasi-pyramidal horn antenna. This paper [30] presents an aperture-shared multi-port rectangular horn antenna with four traditional WR-2S waveguides for 28 GHz. The antenna has a high gain of 16 dBi, low mutual coupling of ≤−20 dB, and sufficient cross-polarization discrimination level at 20 dB in the 27–29 GHz frequency band. The effect of a metallic block on decoupling the feeding port is discussed. Dubrovka, R et al. [31] illustrated a 3-D-printed, 0.3-THz back-to-back horn is presented, made from mating symmetric halves and metalized with gold plate. The horn pair hosts a microfluidic aperture for feeding analytes to characterize solvated proteins. This configuration enhances beam-sample interaction and efficient detection, with initial measurements showing a 2.5 dB increase in signal strength. The study [32] introduces a multi-cut Fresnel-field far-field transformation (MCFFFFT) to measure the single-cut plane pattern of antennas with different wavelength heights. The MCFFFFT, which uses a cylindrical near-field measurement system, accurately reproduces the correct patterns of a horn antenna with 23 dBi at 76.2 GHz when rotated around the boresight direction. Table 3 provides details regarding the specifications of the horn antenna manufactured via 3D printing.

**Table 3:** Specifications of the 3D-printed horn antenna.

Ref.	Freq	Antenna type	Fabrication technique and materials	Application	Advantage	Limitation
[21]	UHF	Rectangular horn antenna	SLM process AlSi10Mg	Astronomical applications in the W band	Benefits areas where application-specific designs are frequently used to optimize system performance and where the best outcomes may need a relatively high level of customization. W band performance is satisfactory.	Even after postprocessing, residual surface roughness in the waveguide section may still be blamed for the bulk of this loss, as evidenced by decibel-level deviations and marginal performance deterioration that may be caused by surface roughness as a result of the SLM process.
[22]	SHF	Circularly polarized horn antenna	Selective laser melting (SLM) 3D printing Aluminum powder	X-band satellite communications	Low in weight and uses fewer materials. Because the SLM 3D-printed horn antenna is manufactured as a single solid, seamless component, no assembly, screws, solder, or other binding materials are required. Furthermore, there is perfect electrical continuity between subcomponents, and all subcomponent alignments are guaranteed with micrometer precision.	The expensive process, surface roughness, and untreated rough metal oxidize and dirties fast due to the natural oxidation of aluminum and particles adhering to the rough, nearly porous surface.
[24]	SHF	Horn antenna	3D printing using powder-bed and inkjet head, metalized via chemical deposition. Polymer, copper, and nickel for metallization	UAV/RPA applications Possibly suitable for flying objects with stringent payload requirements	Rapid prototyping of new designs is less expensive and lighter than traditional metalworking, and it can produce a wide range of 3D designs. Furthermore, the polymer base absorbs little heat and moisture and does not corrode. It can combine different metals using a chemical deposition technique, allowing for adapting the conductive layer's radio frequency characteristic. This gives the antenna design an advantage.	Multiple factors could influence electrical and mechanical performance stability, such as the thermal expansion of different materials, method of metallization, including bends, folds, and edges, density of the printed base, lack of holes and cavities, and roughness of the surfaces.
[25]	SHF	Horn antenna	SLA-based 3D printing Metalized plastic	Mobile SATCOM applications	Flexibility, grating lobe-free, wideband, and low-profile waveguide	Dual circular polarization. The measured axial ratio at the broadside is worse than the simulations.
[26]	SHF	Mode converter horn antenna	FDM PLA was coated with a thin aluminum layer	TEM mode to TE11 mode conversion at 12-GHz frequency. In addition, it can be used as a TM01 to TE11 mode converter.	Design flexibility, accuracy, and cost reduction achieved using 3D printing. The MCA does not have the previous structural issues and can handle large amounts of power.	MCA frequencies are limited due to the limited volume that can be generated using 3D printing and the associated cost.

## 5 RFID antenna

RFID antennas are classified as planner antennae and can be designed in various shapes and sizes, integrated into the RFID reader, or be a separate component. The RFID antenna design is crucial to the performance and efficiency of the RFID system and can affect factors such as read range, accuracy, and data transfer speed.

3D-printing technology recently saw a rising demand in the fabrication of 3D electromagnetic structures. This approach offers various benefits over conventional production processes, including a smaller form factor, less weight, reduced cost, and environmental friendliness.

**Fig. 7:**
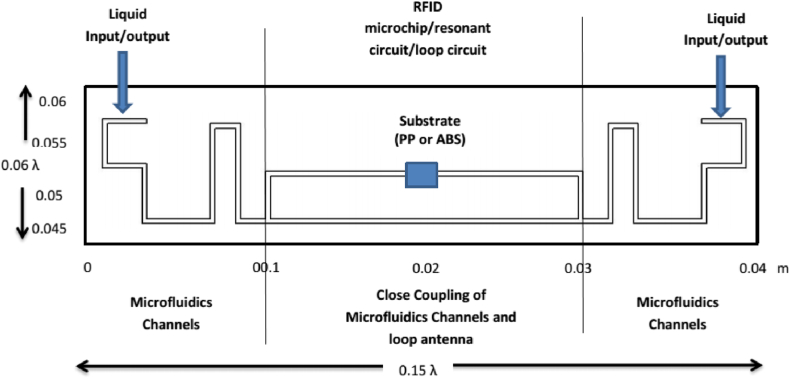
Schematic diagram of the meander-type antenna [30].

Pekgor et al. [33] introduced a new form of 3D-printed polymer for radio frequency identification in an ultrahigh-frequency band based on the liquid antenna. They found that the designed and constructed 3D-printed hybrid antenna may be employed in various embedded systems, such as blood or oil circuits, for chemical and biological sensing. Fig. 7 shows the schematic diagram of the meander-type antenna. The substratum microfluidic channels were designed and 3D printed with an embedded RFID microchip. The resonant circuit and loop antenna were developed based on ALN-9762-WRW model with a short inlay tag by the close-coupling method.

**Fig. 8:**
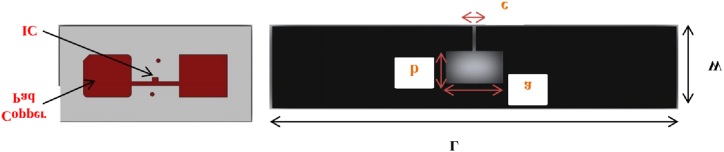
Schematic diagram of the flexible UHF RFID antenna [31].

He [34] suggested a unique manufacturing procedure and evaluated the efficacy of textile-integrated, passive, ultrahigh frequency radio identification devices using 3D printing and embroidery. The antenna was made by 3D printing a stretchy silver conductor directly on an elastic band. The results showed that combining 3D printing and embroidery provides a feasible solution for creating textile-integrated wireless systems. Tu et al. [35] designed and screen-printed a third L-shaped multi-resonator wearable RFID tag with symmetry on the fabric. The antenna frequency response characteristics were tested and analyzed to investigate the impact of a nonuniform conductive layer on signal transmission at higher frequencies. Liu et al. [36] suggested a button-shaped, 3D radio frequency RFID tag that combines 3D printing with inkjet printing. The button design facilitates tag integration with clothing for identification and access management. Fig. 8 presents a schematic diagram of the flexible UHF RFID printed on the stretchable textile to utilize stretchable textile-integrated RFID components. The antenna is quite wide (2 cm), which reduces the impact of imperfections in the print outcome, and the length of the antenna (10 cm) is sufficient to avoid the weaknesses of electrically small antennas in the UHF frequencies from 800 to 1000 MHz. Only one layer of ink was used, and the antennas were cured at 110 °C for 15 min.

Parthiban et al. [37] presented a novel probe-fed circularly polarized patch antenna design on a concave-shaped 3D-printed substrate for sub-GHz ISM band (865–869 MHz) applications. They obtained circular polarization using two pairs of asymmetric slot perturbations. Table 4 delineates the specifications associated with the RFID antennas fabricated through 3D printing.

**Table 4:** Specifications of the RFID 3D-printed antennas.

Ref.	Freq	Antenna type	Fabrication technique and materials	Application	Advantage	Limitation
[33]	UHF	Meander-type	FDM Polypropylene (PP) and acrylonitrile butadiene styrene (ABS)	Hybrid liquid antenna (sensor system). RFID for bio applications	It can be used in embedded chemical or biological sensing systems inside perpetual systems such as blood or oil circuits.	Water absorption may cause irreparable damage. ABS rapidly absorbed the ionic liquid during experimentation, destroying the radiation pattern. Water leaking caused the pattern to be inconsistent.
[34]	UHF	Flexible UHF RFID antenna	3D direct-write dispensing to fabricate stretchable RFID tag antennas on a stretchable textile material, i.e., on an elastic band Stretchable silver conductor (DuPont PE872)	Flexible RFIDs for textile and wearable wireless platforms	Improved wireless performance at a reduced cost	Extreme stretching was too much for the epoxy-glued or 3D-printed antenna-microchip connections.
[35]	UHF	Symmetrical 3D L-shaped multi resonator wearable chip-less RFID tag	Screen printing on fabric as the substrate Black polylactic acid filament	Wearable chip-less RFID applications	Performance augmentation. Future wardrobe management or health monitoring applications have much to gain from a fabric-based chip-less tag with improved resolution and more encoding capacity.	The radio frequency performance of chip-less RFID tags is impacted by technical challenges in managing screen-printing accuracy on fabric.

## 6 Dipole antenna

A dipole antenna is a primary radio antenna comprising two conductive elements, usually metal rods or wires of equal length and separated by a small distance. Dipole antennas are simple, inexpensive, and widely used for various applications, including short-range communications, amateur radio, and TV and FM radio broadcasting. They are known for their omnidirectional radiation pattern. The 3D printing of dipole antennas involves using a 3D printer to create a physical structure for the antenna elements and the transmission line, which allows for the creation of customized, complex, and lightweight antenna designs. 3D printing also enables the integration of the antenna and the transmission line into a single unit, which reduces the number of components and simplifies the installation. Using conductive materials, such as metal-filled filaments or conductive inks, is necessary for the 3D printing functional dipole antennas. 3D printing offers the potential for cost savings and improved performance compared to traditional antenna manufacturing methods.

Swapna et al. [38] proposed a four-port pattern diversity antenna with a 3D-printed, all-dielectric superstrate for a WLAN access point. They found that the dielectric superstrate was given a gain boost of 3.8 dB for antenna 1 and 4 dB for antennas 2 and 3. Meanwhile, Wang et al. [39] reported a high-performance, printed graphene-based antenna. They created a graphene-conductive ink through the liquid-phase exfoliation process. The ink was printed on transferable water paper using the blade printing technique, patterned as a dipole antenna, and then transferred to a target substrate.

**Fig. 9:**
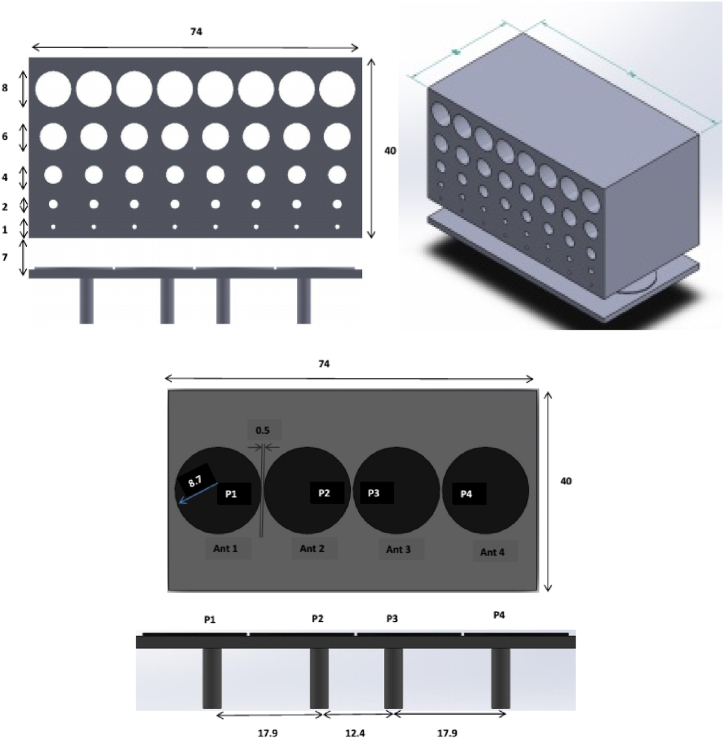
Representation of the four-element folded-type dipole array antenna [35].

In conclusion, the 3D-printed graphene antenna met the Internet of Things application requirements and could replace traditional metallic antennas in specific applications. Fig. 9 displays the representations of the four-element folded-type dipole array. These are circular patch antennas with an individual radius of 8.7 mm (0.15λ) each. The four antennas are coaxially fed with a port-to-port distance of 17.9 mm (0.3λ) and 12.4 mm (0.22λ).

**Fig. 10:**
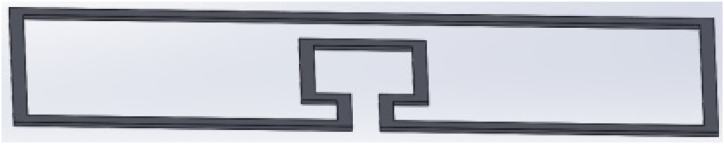
Representation of the dipole antenna [37].

Khan et al. [40] presented a 3D-printed dipole antenna made of a conductive copper filament and successfully printed three ultra-frequency RF-identifying antennas. Phan et al. [41] investigated the bending of a paper-based printed antenna to predict the behavior of an antenna placed in a limited-dimension packaging. Their research began with an angle dipole model comprising two misaligned components for qualitatively analyzing the influence of the angle between the two components and the features of their length on the dipole's radiation qualities. Wen et al. [42] present a novel-type dielectric rod antenna with the merits of low cost and easy manufacturing process. They also introduced a dipole array as a feed source. Fig. 10 depicts a representation of the dipole antenna. After the antenna printing process, NXP UCODE G2iL RFID ICs (integrated circuits) were attached to the 3D-printed dipole antennas using conductive silver epoxy.

Chen et al. [43] investigated a dipole antenna with a 3D diamond structure electromagnetic bandgap and its transmission performance to improve the antenna radiation performance. They used 3D printing to create a diamond structure using an aluminum oxide ceramic. Table 5 outlines the specifications for the dipole antenna manufactured using 3D printing technology.

**Table 5:** Specifications of the 3D-printed dipole antenna.

Ref.	Freq	Antenna type	Fabrication technique and materials	Application	Advantage	Limitation
[38]	SHF	Four-element folded dipole array	FDM for all-dielectric block PLA	WLAN access-point devices	The antenna topology provides beam tilting of 15° and 45° without using phase shifters, as the phase centers of the individual antenna element differ from the ground plane. The all-dielectric 3D-printed metamaterial improved gain at 45° and 15°, resulting in equal gain throughout.	High-dielectric-constant materials have higher manufacturing costs as they cannot be 3D-printed at a low cost; instead, they must be produced using laser technology or milling, followed by a relatively expensive lamination process.
[39]	UHF	Dipole Antenna	Blade printing technique (water-transferable paper) Graphene conductive ink	Internet of Things applications replacing conventional metallic antennas	Scalable manufacturing of graphene-based antennas using a low-temperature, green technique. It is compatible with several IoT protocols, including Bluetooth, 2.4 GHz ANT, and 802.15.4 (Zigbee, Thread).	Individual chips' sensitivity and transmission power impact the antenna's reading range.
[40]	UHF	Dipole antenna	FDM Electrify, conductive copper-based filament	UHF RFID antenna embedded in 3D-printed structures	Reduction in fabrication time and materials.	Similar antenna designs constructed utilizing conventional production processes and materials have substantially greater read ranges.

## 7 Helical antennas

**Fig. 11:**
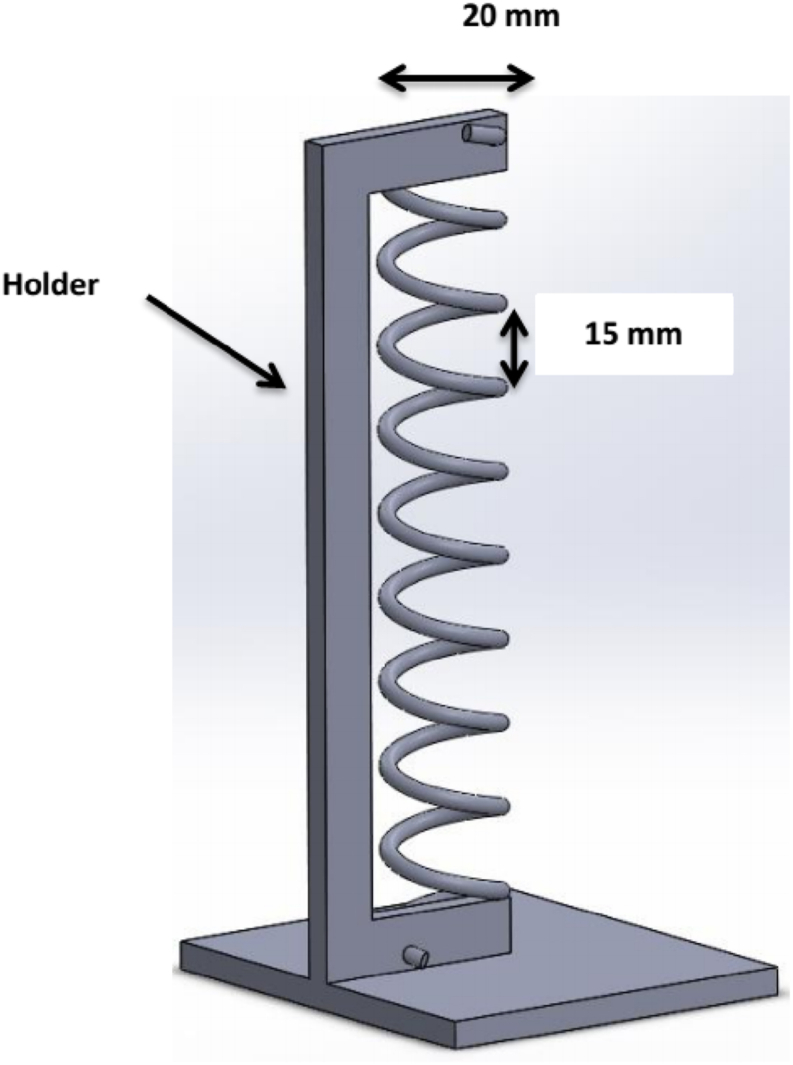
3D CAD model of a circularly polarized 3D-printed helical antenna [42].

Bharambe et al. [44] provided a straightforward procedure for creating intricate 3D helical antennas by vacuum-filling 3D-printed cavities with gallium-based liquid metals at room temperature. A commercial printer was used to create voids by printing a material that resembled sacrificial wax along with acrylic resin. Ghassemiparvin and Ghalichechian [45] manufactured a circularly polarized, 5-GHz helical antenna using 3D printing. They tested the antenna's suitability for satellite communications. Also, they investigated several commercially available dielectric printers and materials for manufacturing, including PC, PLA, and ABS. Moreover, they performed nickel electroless plating, followed by copper electroplating to metalize the final 3D-printed antenna. The geometry of the helical antenna is depicted in Fig. 11, and the antenna is designed in axial mode for C-band (4–6 GHz) at the center frequency of 5 GHz. Table 6 presents the specifications for the helical antennas fabricated using 3D printing techniques.

**Table 6:** Specifications of the 3D-printed helical antennas.

Ref.	Freq	Antenna type	Fabrication technique and materials	Application	Advantage	Limitation
[44]	SHF	Helical antenna	Utilizes an inkjet printing VisiJet M3 Crystal® material and a photocurable dielectric resin to vacuum fill gallium-based liquid metals for metallization into 3D printed cavities.	RF communication application	Using a straightforward method, it can quickly prototype 3D embedded antennas and other microwave parts with metallic conductivity at ambient temperature.	The overall efficiencies of the antennas created using this method are lower than what is anticipated of a comparable antenna manufactured using standard materials (e.g., copper and engineered RF composites).
[45]	SHF	Circularly polarized 3D-printed helical antenna	FDM for antenna fabrication, nickel electroless plating, and copper electroplating for metallization. PLA and ABS, metalized with nickel and copper	Satellite communications	Deliver quick and inexpensive complicated structure prototypes and manufacture. High gain, circular polarization, and straightforward construction.	Gain reduction and differences between measured and simulated results due to helical antenna deformation and shrinkage due to high temperature during the metallization process

## 8 Yagi–Uda and Bowtie antenna

**Fig. 12:**
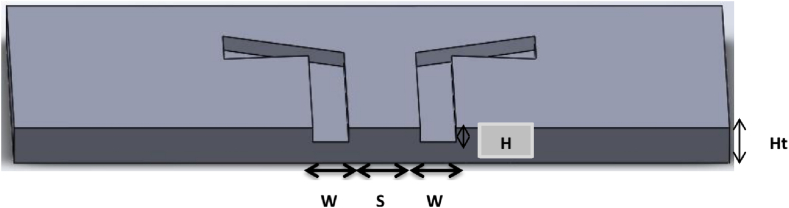
3D CAD model of the Bowtie antenna structures [43].

Aydın and Torun [46] studied the microwave performance of a conductive substrate fabricated using 3D printing technology for biomedical imaging applications. The acquired results indicated that the 3D-printed PLA/carbon antenna was well-suited for biomedical imaging systems. Colella et al. [47] Utilized FDM, 3D printing to create a Yagi-Uda Antenna with Fully 3D-Printed Bow-Tie Elements. The proposed antenna is shown in Fig. 12, which is pre-optimized to see the effect of substrate materials first. The performance of the proposed antenna was evaluated from 1 MHz to 3 GHz, and S11 data was obtained. Table 7 outlines the specifications for the 3D-printed Yagi-Uda and Bowtie antennas.

**Table 7:** Specifications of the 3D-printed Yagi–Uda and Bowtie antenna.

Ref.	Freq	Antenna type	Fabrication technique and materials	Application	Advantage	Limitation
[46]	UHF	Bowtie antenna structures	FDM Pure PLA, PLA/copper, and PLA/carbon	Biomedical imaging applications.	Its improved performance, low cost, and compact size make it suitable for bio applications.	It is more costly than FR4 due to the lack of large-scale manufacturing.
[47]	UHF	Yagi–Uda	FDM PLA (Electrifi)	WiFi applications for UHF antennas	Using 3D printing to prototype effective UHF antennas and RF equipment quickly.	The bare minimum thickness of a single printed line must be considered. When developing electromagnetic structures, a 15% increase in the effective thickness of the extruded line must be carefully considered.

## 9 Lens antenna

3D printing produced lens antennas for millimeter-wave frequencies with equivalent performance to those manufactured using traditional techniques. Saghlatoon et al. [48] introduced a low-cost, multilayered, electrically small dielectric lens to increase the maximum radiation gain and the side lobe level of a directional antenna similar to a horn antenna using current AM filling processes. Instead of using separate materials for each layer, they used the same material for all layers to obtain the best performance.

Belen and Mahouti [49] used the 3D printing technology to prototype nonuniform substrate dielectric lens antennas. The proposed fabrication method was appropriately compared to the conventional fabrication process. Mahouti et al. [50] employed 3D-printing technology to develop a high-performance, inexpensive X-band multilayered cylindrical dielectric lens prototype. The suggested 3D-printing technology permits a high-performance design. Moreover, it offers a rapid, cost-effective, and efficient prototype procedure applicable to various microwave devices. Zhang et al. [51] developed a surface roughness modeling method with a dielectric metallic cooperatively 3D-printed V band lens antenna. The entire potential of the metallic and dielectric 3D-printing processes was used in this method to regulate the lens antenna.

Meanwhile, Anwar et al. [52] presented a novel, 3D-printed dielectric lens for enhancing the gain parameters. The lens was fabricated using the FDM technology, a cost-effective and efficient 3D-printing technique. Liu et al. [53] presented a 26-GHz 3D-printed cylindrical lung burg lens antenna. The proposed antenna consisted of a feeding waveguide, a 3D-printed cylinder, and a pair of printed metal grids adhered to the cylinder surface. The experimental findings illustrated the feasibility and applicability of the proposed antenna. Zhang et al. [54] presented two flat-graded index lenses. The first is a thick lens created and produced using 3D-printing technology. The second is a thin-dial dielectric lens built and constructed utilizing state-of-the-art artificially made dielectric materials. Jeong and Ghalichechian [55] showed that a planner and thin-grooved Fresnel lens have a high potential for improvement in millimeter-wave communication, imaging systems, and wireless power transmission applications. The design and production processes that used a single material were also presented. Isakov et al. [56] stated that gradient refractive index (GRIN) materials are particularly relevant to various applications in which the transformation of optic concepts may be used to create improved photonic and microwave devices. They designed and built a 3D-printed GRIN lens at Ku-band microwave frequencies and characterized and tested it. They achieved higher antenna directivity while noticeably reducing the physical length by combining the GRIN lens with an open-aperture horn.

## 10 Reflector antenna

He and Tentzeris [57] presented the design and manufacturing of an entirely 3D-printed dish antenna with an integrated feeding. They concluded that a 3D-printed antenna is considerably superior to a standard dish antenna with separate feeding antenna and cable components.

Table 8 provides the specifications for the dish antenna manufactured through 3D printing methods.

**Table 8:** Specifications of the 3D-printed dish antenna.

Ref.	Freq	Antenna type	Fabrication technique and materials	Application	Advantage	Limitation
[57]	SHF	Parabolic dish shape	Stereolithography (SLA) printing Formlabs clear photoresin	Long-range communications	Feeders and connectors are integrated with the dish antenna construction through 3D printing, resulting in low costs, simple manufacturing, and easy deployment while providing performance comparable to the standard machined dish antenna.	Specific design considerations must be considered when 3D printing the antenna, such as supporting structures that result from the fabrication process and, when removed, can leave minor imperfections on the dish surface that could reduce efficiency.

Zhang [58] reported a revolutionary AM technique for quickly prototyping a low-cost, lightweight dielectric resonator reflectarray utilizing FDM 3D printing. The reflectarray was built using 625 3D-printed dielectric resonator components to adjust the reflected phase throughout the reflector surface. This process might also fabricate high-gain millimeter-wave antennas with considerably decreased labor time and material costs. Furthermore, Mahouti et al. [59] showed the design and implementation of a 3D-printed alumina-based ceramic substrate reflectarray antenna with numerical calculation and experimental measurement findings. The suggested antenna showed good potential for satellite communication applications in the X band. Belen et al. [60] used 3D printing to develop and realize a wideband, flat-gain, multilayer, nonuniform reflectarray. The proposed optimized design was accomplished in two stages: a 3D CST microwave studio–based multilayer perception neural network model establishes the reflection phase characteristics of the unit element, which is an accurate continuous function of the geometrical design specifications and the dielectric constant.

Zhu et al. [61] proposed a K/Ka high-gain aperture-shared multibeam parabolic reflector antenna. They observed that this type of antenna performed a 2D beam scanning from a shared single parabolic reflector by introducing off-focal feeds. Carkaci and Secmen [62] demonstrated using 3D printing and conductive paint technologies to realize a Ku-based feed system for reflector antenna satellite communication systems. Tariq, S. et al. [63] presented featuring an annular ring, a wheel-shaped concentric structure, and two circular stubs for phase angle coverage. It offers 576° phase variation, 26.6 dBi peak gain, 49.24% aperture efficiency, and 31.5% gain bandwidth. The paper [64] unveils a pioneering Reflectarray design inspired by origami, employing a Hold-Down Release Mechanism and spring-loaded hinges for self-deployment, ensuring flatness accuracy with a unique hinge-latching system. Tailored for compact satellite platforms weighing 50–100 kg, the OSS Reflectarray boasts an 8:1 footprint stowage ratio. Wu et al. [65] proposes a 1-bit reconfigurable reflectarray antenna using liquid metal for beam steering and consists of 3 layers, with a translucent resin casing and a thin dielectric layer. The 1 bit state fills the top casing with liquid metal, while the bottom casing is air and it generates scanning beams within ±40°, with a maximum gain of 24.08dBi and an aperture efficiency of 20.36%. Yang et al. [66] presents a low-RCS ReflectArray (RA) antenna design using a Frequency Selective Surface (FSS) absorber and a reflective metasurface which has an aperture efficiency of 42.7%, a gain of 25.4 dBi in the X band, and a fractal bandwidth of 120%. It is suitable for integrated stealth communication systems. Ali et al. [67] explore the potential of vortices, or orbital angular momentum (OAM) beams using low-cost and high-gain dielectric reflectarray antennas (RAs) at the terahertz band. It proposes a 3D beam-steering or OAM multiplexing paradigm using tilted multiple off-centered OAM beams with different modes. Shabbir et al. [68] proposes an antenna with a slotted square patch and quad meander delay-lines to increase the reflection phase range. The antenna offers a 25 dBi gain and 52.8% aperture efficiency for a 10 GHz frequency band, with minimal cross-polarization and side-lobe levels. Hassan et al. [69] introduce a graphene-based reflectarray antenna with dimensions of 3.75 mm × 3.75 mm and 40 hexagonal-shaped unit cells with a reflection coefficient phase range of 525° placed on a silicon dioxide substrate with a horn antenna at the focal point achieving maximum gains of 18.7, 19, and 17.7 dB at different frequencies. Herhil et al. [70] present electrodynamic simulation and measurement results for an X-band offset reflectarray with 460 mm diameter on thin dielectric substrates. The antenna's main characteristics were optimized with a gain of 27.7 dB and a −3 dB main-lobe beamwidth. Imaz-Lueje et al. [71] present a reflectarray antenna for satellite mega-constellations, featuring a shaped-beam isoflux pattern for constant power flux on Earth's surface using a unit cell with two stacked microstrip patches and a ground plane, providing over 360° phase-shift. The generalized intersection approach optimization algorithm synthesizes the pattern in a 2 GHz bandwidth in Ku-band, showing low-profile capabilities. Hosseini and Oraizi [72] introduces a new graphene cell-cluster, a geometrical configuration of identical unit-cells used in a reflectarray with a middle layer of metallic patch. Fourier-optics and aperture field estimation is used to investigate the effect of cell-cluster dimensions on far-field radiation patterns, resulting in enhanced design accuracy. Cui et al. [73] present a 3-D-printed dielectric reflectarray antenna with one-shot deployability and wide-angle beam-scanning ability, featuring a “kirigami”-inspired two-stage snapping-like element structure fabricated using stereolithography and Formlabs Flexible 80A photopolymer, demonstrating high-performance for 5G and satellite communication applications. Guo et al. [74] propose an ultra-wideband 3D-printed dielectric reflectarray antenna (DRA) using a photosensitive resin therm1220. The dielectric reflectarray unit (DRU) has a dielectric constant 3.97 and can be adjusted through size adjustment. The gain ranges from 25.8 to 28.9 dBi, with a 3 dB gain bandwidth of 32.1%. Low-cost antennas for wide-angle beam scanning in the mm-Wave band are crucial for future terrestrial and satellite communication systems. Phased array antennas are popular due to their low profile and mature manufacturing process, but their power consumption and large active components remain drawbacks. Spatially fed antennas like reconfigurable reflectarray or transmit arrays overcome these issues [75]. Microstrip implementations of reflectarrays are narrow-band and affected by conductor and surface wave losses. Grounded dielectric layers offer improved performance and flexibility. Designing reflectarrays is challenging due to numerous variables and multiple requirements. An alternative approach uses deep learning to construct unit element representations, reducing the total RA optimization cost to a few hundred samples. This approach is validated using 3-D pyramidal-shaped elements and discussed for experimental verification by Mahouti et al. [76].

## 11 Vivaldi antenna

Gjokaj et al. [77] presented the design, production, and testing of an ultra-wideband Vivaldi antenna that can take advantage of the low-loss characteristics of a substrate-integrated waveguide-like structure. The antenna had a 10.3-dB maximum gain and 20-GHz bandwidth. Moreover, Clower et al. [78] studied an additively manufactured Sierpinski tetrahedron–based prefractal antenna design and used the finite element technique modeling to simulate the Sierpinski tetrahedron. They also performed an experimental examination to demonstrate its potential for wideband communication. In conclusion, the process offers the most benefits for producing antenna products.

Bakytbekov et al. [79] examined a printed system-on-package-based 3D cube triple band cantor fractal receiver. The box carrying the antenna contained a multiband matching network and a rectifier circuit. The acquired results and the low cost of the recommended design made it a practical option for IoT device manufacturing. On the contrary, combining SoP with AM ensures reduced cost and efficient use of space.

Table 9 details the specifications of the Vivaldi antenna fabricated using 3D printing technology.

**Table 9:** Specifications of the 3D-printed Vivaldi antenna.

Ref.	Freq	Antenna type	Fabrication technique and materials	Application	Advantage	Limitation
[77]	SHF	Vivaldi antenna	VeroWhitePlus, a UV-curable polymer, is used in polyjet 3D printing utilizing an Objet Connex 350 printer.		The 3D-printed products' substantial material losses can be overcome	Differences in measured and simulated return gain findings, particularly above 14 GHz.
[78]	Wideband multifrequency response	Third-order Sierpinski tetrahedral antenna	FDM ABS spin-coated with graphene flakes. Graphene-impregnated PLA filament	Devices with high performance, enhanced bandwidth, and small form factor may be used to miniaturize spacecraft, drones, and UAVs and have applications for wireless energy harvesting and cellular communication systems.	Rapid prototyping and the construction of higher-order complex prefractal structure antennas. It offers enhanced gain and multiband performance at a low cost, with high dimensional precision. Moreover, it exhibits corrosion resistance and low weight.	The material used has lower electrical conductivity than copper.
[79]	Wideband multifrequency response	Cube triple band Cantor fractal rectenna 3D cube-shaped fractal antenna	Screen printing using Kapton tape as a mask. Conductive silver paste	Harvesting RF energy for IoT devices; it can concurrently collect RF energy from the GSM900, GSM1800, and 3G 2.1-GHz bands.	Compact size and reduced cost using AM technology and the system-on-package concept. It is a multiband RF energy harvester due to its fractal antenna shape; thus, it can be utilized to power IoT devices.	The considerable discrepancies between the observed and simulated findings at high frequencies might be attributed to the looseness of the 3D-printed material, which increases loss at higher frequencies.

## 12 Wire antenna

Memon et al. [80] used inkjet printing on Kodak photo paper to create two miniaturized wire antennas utilizing a 3D space-filling Hilbert curve fractal geometry. The suggested antennas had the 3D dimensions of an 8-cm^3^ box and operated at even higher frequencies of 238 and 147 MHz.

## 13 Other antenna types

AM is used to fabricate other antennas and communication devices. For example, Czelen et al. [81] presented a tiny electronically steerable parasitic array radiator antenna. They embedded the antenna's active and passive elements into the PLA material during fabrication using the 3D-printing technology, which resulted in a size reduction. They showed a good agreement between the simulation and experimental results. The findings revealed that the printed antenna performed well and matched the simulation results. Lou and Wu [82] demonstrated a 3D-printing method to fabricate stereo antennas using dual-material FDM and selective chemical plating.

Karthikeya et al. [83], constructed a millimeter-wave antenna at 28 GHz for 5G base stations and mobile terminals. A 3D-printed radome with a small pattern diversity module for millimeter-wave 5G base stations was also presented. The constructed radome maximizes the gain while having a small physical footprint. For millimeter-wave applications at 28 GHz, Sabri et al. [84] built a highly directive dual-beam waveguide-slotted antenna using 3D metal–printing technology. The dual-beam directional patterns may also double the coverage. The antenna performance was investigated, and the experimental results agreed well with the simulation. Asci and Yegin [85] proposed a novel 3D-printed wideband and low-cost bull's antenna for Ku-band applications. The proposed antenna spanned the complete Ku-band satellite communication frequency from 10.5 to 14.5 GHz. Furthermore, 3D-printing technology and conductive painting were used to fabricate the antenna.

Using the AM technology, Asci and Yegin [86] aimed to analyze the effect of several types of groove shapes around the aperture antennas to examine their performance. They constructed a waveguide slot-fed dual-capacity aperture antenna with grooves and investigated the groove shape's effect on the Ku-band's antenna performance. García‐Vigueras et al. [87] proposed a compact dual-polarized directive radiator with a challenging prototype made possible through AM. SLA was used to achieve monolithic implementation, followed by metal coating. The antenna offered a high-frequency performance at a lower cost and weight and allowed for simultaneous dual-channel operation.

In conclusion, the proposed antenna could be utilized in platform-restricted applications, such as CubeSat inter-satellite communications. Mohassieb et al. [88] studied the effects of varying the drop spacing of ink during inkjet printing on the printed films' conductivity and the antenna's characteristics. Hence, they used silver nanoparticle ink on polyethylene terephthalate and Epson paper substrate to develop and print a low-profile wideband waveguide-fed monopole antenna operating at 20 GHz. They also evaluated the effect of altering the ink drop spacing on the conductivity of the printed films and the antenna variables, which were thoroughly analyzed using numerical models and tests. Shamsinejad et al. [89] presented a unique 3D conformal annular slot antenna type. It was patterned over a 3D-printed cubic package with a conductive surface to produce a horizontally polarized omnidirectional radiation pattern in the azimuth plane.

A cubic antenna package at 2.4 GHz was built and constructed to validate the wireless sensor network application concept. Lee et al. [90] suggested a flush-mountable 3D-printed tapered cavity–backed wideband antenna for UAV applications. The antenna comprised a monocone and an exponentially tapered cavity printed via 3D printing. They looked at the performance loss caused by the resonance in a conventional cavity mitigated by narrowing the cavity. Yu et al. [91] proposed a probe-fed, open-ended waveguiding antenna. It was entirely integrated using a 3D-printing method. Traditional processing methods, such as computer numerical control machining, need postprocessing assembly when fabricating the complicated waveguide structure. The proposed design shows how an all-in-one waveguide filtering antenna may be expanded to include various waveguide components. Mirzaee and Noghanian [92] concentrated on the high-frequency characterization of the wood fill filament for 3D printing and its practicality in fabricating small-sized antennas using the AM technique. They used three different conductive materials for the antenna's conductor components. Cao et al. [93] present a 3D printed multi-beam orbital angular momentum antenna featuring a pillbox BFN, slotted waveguide antenna array, and full-dielectric phase elements. The prototype achieves five +1 mode beams with a ±20° 3 dB beam coverage range. Koziel [94] investigated surrogate modeling techniques that are crucial in antenna structure design due to high costs. Performance-driven modeling and nested kriging improve predictive power, which combines constrained modeling with a new PDRN surrogate, enhancing accuracy without increasing dataset cardinality. Persad [95] discusses the significant R&D and application areas for 3DP, examines mainstream 3DP technologies, explores research incorporating traditional printing, proprietary methods, and composite 3DP methods, and classifies 3DP-built EM structures. Rice et al. [96] discussed a biocompatible high-contrast low-loss antenna (HCLA) is designed for efficient into-body radiation in medical telemetry, sensing, and imaging applications that operates across the 1–5 GHz bandwidth and is loaded with a high-contrast and low-loss dielectric for improved directivity and gain fabricated using stable, low-loss materials, allowing for repeatability and consistency.

## 14 Dielectric substrate

Francis and Jain [97] characterized the material characteristics of the nanocomposites and created a PCN that demonstrated an increase in permittivity values compared to a pure polymer. The ionic nature of the nanocomposite and the fused network of the nanocomposites developed during the 3D printing contribute to improving dielectric characteristics. Prashantha and Roger [98] created conductive polymer nanocomposites via 3D printing. The suggested approach had an additive multilayer deposition of the polymeric nanocomposite based on PLA and graphene, which was printed using a low-cost commercial tabletop 3D printer. However, the suggested material has a clear advantage when employed in 3D-printed structures because including multifunctional graphene considerably enhances the characteristics of the resultant nanocomposite. Ratni et al. [99] presented a beam steerable Fabry–Perot cavity antenna design based on a phase-modulated partly reflecting surface. A 3D-printed index-modulated dielectric substrate was utilized to incorporate the phase modulation. Table 10 presents the specifications for the dielectric substrate fabricated using 3D printing techniques.

**Table 10:** Specifications of the 3D-printed dielectric substrate.

Ref.	Freq	Antenna type	Fabrication technique and materials	Application	Advantage	Limitation
[97]	100 KHz–1 MHz	3D-printed polymer dielectric substrates	FDM nanoclay (cloisite 30B) and ABS pellets Polymer clay nanocomposites	Industries that need dielectric substrate materials for manufacturing devices used in electronics and communications	Improved polymer permittivity as compared to ordinary pristine polymer	Nanoclay inclusion may considerably modify the dielectric characteristics of polymer nanocomposites.
[98]		3D-printed conductive polymer nanocomposites	Fused deposition modeling (FDM) PLA and graphene	Applications related to tissue engineering as well as bioelectronics and biosensors.	Improved mechanical and thermomechanical properties of neat PLA as a result of the addition of graphene nanoplatelets, which considerably improve the properties of the resulting nanocomposite and has a distinct advantage when used in 3D-printed structures.	Increased electromagnetic interference EMI that can be attributed to the increase in the conductivity of the nanocomposite material

## 15 Potential of using antennas in civil engineering applications

Several antennas are designed for civil engineering applications. Salama and Kharkovsky [100] proposed an embeddable rectangular microstrip patch antenna with a receiving mode approach to investigate the performance of the embedded antenna. When embedded in concrete, the antenna is designed to operate at ∼2.5 GHz. It can be used for wireless power transmission through building materials, such as concrete, and for material characterization. Castorina et al. [101] presented an antenna design that can be embedded in concrete. This study describes an embedded circularly polarized patch antenna for the wireless monitoring of concrete structures. A real-time indoor localization (RFID) system is created to monitor Fang et al.'s building sites [102]. The suggested method locates construction workers on a BIM model using an RFID antenna and provides real-time visualization on multiple devices through a cloud server for remote monitoring. The reader sends the information gathered by reading tags using an RFID antenna to a host computer for additional processing and analysis. The system may cover spaces with various geometrical configurations and numerous stories. Another civil engineering application was proposed by Moosazadeh et al. [103]. This study adopted an antipodal Vivaldi antenna (AVA) based on a conventional AVA (CAVA) design. The impedance bandwidth of the AVA antenna ranged from 1.65 to 18 GHz. The front-to-back ratio was 42 dB at 13.5 GHz. An AVA antenna was used to detect the voids inside the concrete beams.

Lai et al. [104] presented researchers' and practitioners' efforts toward using ground-penetrating radar (GPR) antennas in 30 years. GPR antennas have been used in various applications, from locating and testing to imaging and diagnosis. Ref. [104] presented the applications of GPR antennas in aging buildings, pavements, bridges, tunnels, infrastructure, and underground utilities to assess the as-built status of the existing structures and determine their current deterioration state. Table 11 lists some of the GPR antenna applications in civil engineering. The table also presents the type of investigation conducted by researchers and practitioners, the antenna frequency used in the investigation, and the type of civil engineering application. These GPR antennas can be produced in various shapes and sizes using 3D-printing technologies, including FDM, SLA, and SLS. Furthermore, the utilization of ceramic substrates in antenna manufacturing can be significantly enhanced by integrating 3D printing technology [105]. Additionally, when it comes to printing antennas at the microscale, incorporating nanomaterial composites becomes essential, providing valuable support and advancements in implementing 3D printing technology [106,107].

**Table 11:** Selected applications of GPR antennas in civil engineering.

Type of investigation	Antenna frequency	Application	Reference
Locate steel reinforcement to assess the seismic vulnerability of a building	1600 MHz	Buildings	[108]
Detect local weakness points in the floor, original power lines, and water conduction of a historical building in Spain	400 and 900 MHz	Buildings	[109]
Detect cracks penetrating into the thickness of the ashlars, pulse penetration into masonry walls, and detect cultural heritage buildings	1.6 GHz	Buildings	[110]
For a concrete structure in Korea, provide photographs relating to the reinforced steel bars and faults, such as voids (voids beneath the reinforcing steel bars could not be found).	1200 MHz	Buildings	[111]
Detect voids and cracks before and after cement injection in a tower building in Hungary	400 and 900 MHz	Buildings	[112]
Detect local weakness points and cavities in floors and water conduction in a school structure in Turkey	2 GHz	Buildings	[113]
Detect the position of dowels and tie bars	1.6 and 2.6 GHz	Concrete pavement	[114]
Determine concrete pavement thickness	2 GHz	Concrete pavement	[115]
Determine pavement thickness and detect air voids in reinforced concrete pavements	1.5 GHz	Concrete pavement	[116]
Detect cracks in road pavements	250 and 1000 MHz	Road pavement	[117]
Detect cracks in asphalt pavements	1 GHz	Road pavement	[118]
Determine highway roadbed damage	200 and 400 MHz	Highway pavement	[119]
Determine asphalt pavement thickness	200 MHz–3 GHz	Road pavement	[120]
Monitor moisture condition in the subgrade and road base of asphalt pavements	900 MHz	Road pavement	[121]
Determine layer thickness and detect pavement roughness of a motorway	2 GHz and 400 MHz	Road pavement	[122]
Roadway structure evaluation of a highway	0.5–6.5 GHz	Highway pavement	[123]
Detect damaged rebar and moisture ingress of a road bridge	2 GHz	Road bridge	[124]
Track cracks and corrosion associated with reinforcement bars	2 GHz	Concrete bridge	[125]
Explore inadequate concrete covers	1.6 GHz and 2.6 MHz	Concrete bridge	[126]
Detect the deterioration of surface concrete	1.5 GHz	Concrete bridge	[127]
Locate the rebar and measure the depths of the cover, post-tensioning cable trajectories, and “T” bridge girders.	2 GHz	Concrete bridge	[128]
Determine the liner thickness of a tunnel	900 MHz	Tunnel	[129]
Regular inspection of railway tunnel: detect deformations and determine liner thickness	300 MHz	Railway tunnel	[130]
Determine grouting layer thickness and the presence and distribution of any damage	800 MHz	Metro	[131]
Inspect and identify buried plastic pipes	1.5 GHz	Underground utilities	[132]
Locate utility, shape, and orientation	200 MHz	Underground utilities	[133]
Location accuracy of buried pipes	250 and 700 MHz	Underground utilities	[134]
Locate subsurface utilities	400 MHz	Underground utilities	[135]

## 16 Uses of antennas in chemical applications

Antennas are utilized in various chemical applications. One of their primary applications in the chemical industry is level measurement in tanks and vessels containing chemicals [[136], [137], [138], [139], [140]]. An accurate level measurement is essential to ensure an efficient operation and prevent overflows or spills. Different types of antennas, such as microwave, ultrasonic, and radar, are used for level measurement. These antennas can send and receive signals that determine the chemical level in the tanks and vessels [[141], [142], [143], [144], [145]]. Antennas are also used for wireless communication between equipment and sensors in chemical-processing plants. This helps in the remote monitoring of various chemical processes that can significantly improve the efficiency and safety of the plants [[146], [147], [148]]. Wireless sensor communication can provide real-time data, allowing quick responses to any issues. This can also prevent costly downtimes and improve the plant's overall productivity [149,150]. Leak detection is another important application of antennas in the chemical industry.

Antennas can be used to detect leaks in pipelines, which is essential for ensuring the safety of workers and preventing environmental damage [151,152]. Electromagnetic wave methods are commonly used for leak detection. Antennas can also be used to remotely monitor pipelines to detect any potential issues before they become significant problems [[153], [154], [155]]. In 1999, Mirshekar-Syahkal [156] reported using antennas to determine the dielectric properties of liquids. Due to their geometric structure, lightweightness, cost-effectiveness, and applicability, microstrip patch antennas are commonly used as sensors [157] for chemical liquid detection. Antennas have been used for ethanol detection [158] because the ethanol concentration may reach toxic levels during fermentation and distillation. Karatepe et al. [159] developed multipurpose liquid sensors based on patch antennas to be used for the multitask sensing of liquid mixtures.

Swearer et al. [160] developed antenna–reactor complexes that increased photothermal heating near catalytically active surfaces, thereby allowing light-induced photocatalysis at specific wavelengths, which enables specific chemical reactions and reaction pathways.

Antennas play a critical role in RFID tracking for chemical industry applications. RFID tracking can be used for inventory management, asset tracking, and process monitoring [161]. Antennas enable the RFID system to communicate with the tags attached to chemicals, containers, and equipment. RFID tracking in the chemical industry can help streamline inventory management and enhance supply chain visibility. It can also improve safety by enabling real-time tracking of hazardous materials and equipment.

Furthermore, RFID tracking can monitor the movement of chemicals and ensure that they are stored and transported safely [162]. Moreover, the adaptability and integration into the construction field by incorporating 3D-printed multilayered sandwich panels [163,164] enhances structural robustness and facilitates safety monitoring systems, conveying an advanced layer of caution and resilience to construction practices. The antennas used for RFID tracking in the chemical industry must be designed to withstand harsh environments, such as those found in chemical plants, where they may be exposed to chemicals, extreme temperatures, and humidity [165]. In summary, antennas play a crucial role in chemical processing plants' safe and efficient operation. From level measurement to wireless communication and leak detection, antennas have various applications that can considerably improve the overall performance of chemical-processing plants.

## 17 Conclusions

3D-printing technology is increasingly used in developing and manufacturing antennas owing to their numerous advantages. For example, it enables the production of complex antenna geometries that can enhance the performance of antennas by increasing gain, bandwidth, and radiation efficiency. 3D printing can create customized antennas that can be tailored to specific applications. This is particularly useful in complex geometries and low-volume manufacturing. The production of intricate 3D printed antenna designs that are otherwise difficult or impossible to manufacture using traditional manufacturing methods. Besides, reducing lead time can significantly reduce the lead time and cost of manufacturing antennas. This is particularly true for prototypes, where design iterations can be quickly and easily produced without expensive tooling. Lightweight and compact antennas are another advantage that can be particularly useful in mobile applications where weight and size are critical factors. A wide range of materials for additive manufacturing, including conductive materials, can be utilized to create conductive structures within the antenna. This can improve the performance of the antennas. The 3D printed antenna can facilitate the integration of different components within an antenna, including filters, amplifiers, and connectors, into a single unit, thereby reducing the overall size and complexity of the system. Moreover, rapid prototyping assists and accelerates the development process and reduces the time to market.

## Data availability statement

There is no data associated with this article.

## Ethics declarations

Review and/or approval by an ethics committee was not needed for this study because it is a technical review paper.

## Funding disclosure

UAE University funded this paper.

## CRediT authorship contribution statement

**Muthanna Aziz:** Writing – original draft, Data curation, Conceptualization. **Amged El Hassan:** Writing – original draft, Methodology. **Mousa Hussein:** Supervision, Investigation. **Essam Zaneldin:** Resources, Data curation. **Ali H. Al-Marzouqi:** Visualization, Supervision. **Waleed Ahmed:** Writing – review & editing, Writing – original draft, Supervision, Resources, Investigation, Funding acquisition.

## Declaration of competing interest

The authors declare that they have no known competing financial interests or personal relationships that could have appeared to influence the work reported in this paper.
